# A practical framework for large-scale neuroimaging data collection in clinically diverse populations: experience from the Stanford Brain Stimulation Lab

**DOI:** 10.3389/fnins.2026.1849837

**Published:** 2026-10-05

**Authors:** Azeezat Azeez, Ethan Makarewycz, Natalie Feng, Wiebke Struckmann, Jen Lissemore, Noriah D. Johnson, Malvika Sridhar, Andrew Geoly, Ruben Krueger, Brendan Wong, Seigo Ninomiya, Lauren Crowe, Derrick Matthew Buchanan, Sumaiya Ahmed, Cammie Rolle, Nolan Williams

**Affiliations:** 1 Brain Stimulation Lab, Department of Psychiatry and Behavioral Sciences, Stanford University, Palo Alto, CA, United States; 2 Department of Radiological Sciences, Stanford University School of Medicine, Stanford, CA, United States

**Keywords:** clinical research workflow, MRI study design, neuroimaging research logistics guidelines, reproducible MRI data, systematic collection of MRI data

## Abstract

Novel neuroimaging technologies and data science techniques have allowed for large-scale applications of neuroimaging acquisition in clinical research, particularly within the fields of interventional psychiatry, neuropsychiatry, and precision medicine. These developments have enhanced our understanding of psychiatric and neurological disorders and highlight the need for optimized protocols that support scalable, high-quality neuroimaging data acquisition. This paper aims to describe a protocol for systematic MRI data collection in clinical trials, including details for subject recruitment, meticulous equipment calibration, rigorous real-time quality control of imaging data, standardized documentation (SOP, hardware and software logs), consent workflows, and personnel training. While existing MRI review papers largely focus on analytical methods, safety protocols, and results of large consortium efforts, few publications detail specific operational protocols of MRI data collection that allow for consistent, high-impact neuroimaging research including participant recruitment, staff training, and practical scanning considerations. The operational protocols presented here are designed to facilitate reproducible, high-quality neuroimaging acquisition across clinically diverse populations, with a particular focus on functional MRI. Importantly, many of the recommendations described are grounded in practical experience rather than prescriptive guidelines alone. They represent lessons learned through years of iterative refinement, troubleshooting, and trial-and-error while conducting thousands of MRI scans in clinical research settings. By sharing these operational insights, we hope to provide researchers with practical strategies that can reduce common challenges and accelerate the development of robust neuroimaging workflows.

## 1 Introduction

Advances in neuroimaging technology have improved our understanding of the neurobiological presentation of psychiatric and neurological disorders. In parallel, over the past thirty years, research trials are more regularly employing imaging, leading to an influx of imaging data and presenting the opportunity to apply data science techniques (Rezaei et al., 2023). Psychiatry as a medical discipline has also evolved to include sub-fields like interventional psychiatry, neuropsychiatry, and precision medicine. Within these fields, novel non-pharmacological approaches to treatment, often utilizing imaging methodologies, are expanding our idea of what it means to treat patients, providing the promise of hope to many who are suffering. These converging factors give the Stanford Brain Stimulation Lab (BSL) the unique opportunity to leverage large amounts of human imaging data to improve the field of neuroscience and interventional psychiatry (Williams et al., 2014).

Current neuroimaging literature has largely emphasized methodology for acquisition parameters (Bollmann and Barth, 2021; Brown and Semelka, 2011), preprocessing pipelines (fMRIPrep, MRIQC; Provins et al., 2023), data standardization (BIDS) and guidelines and reproducibility of computational workflows. While plenty of MRI and fMRI review papers exist, such as the Human Connectome Project (Glasser et al., 2016), the Autism Brain Imaging Data Exchange Initiative (Di Martino et al., 2014), the Alzheimer’s Disease Neuroimaging Initiative (Jack et al., 2008), and the UK Biobank (Miller et al., 2016) detail the analytical methods (Hay et al., 2022), safety protocols, and results of large consortium efforts, few present operational and logistical processes of MRI data collection in clinical research settings within large single-sites for immediate translational impact (Alfaro-Almagro et al., 2018; Bartnik et al., 2024; Koch et al., 2025). Understanding key processes enables lasting scientific impacts, including extensive training of personnel, meticulous equipment calibration, rigorous real-time quality control measures, detailed documentation (SOP, hardware and software logs), and establishing consent workflows is essential for future neuroimaging research. By utilizing these strategies and implementing good scientific practices, researchers can maximize the quality and quantity of usable clinical neuroimaging data.

Adopting strong data acquisition practices in clinical neuroimaging research is vital for this evolving field of study. Recommendations and logistical advice on the collection of human subjects are not only helpful to research laboratories in the Global North but also to those in the Global South, where the infrastructure for gathering neuroimaging data is lacking or nonexistent (Eke, 2024; Pickersgill, 2021; Lock et al., 2024). The quality of collected data is paramount to the accuracy of mechanistic interpretation and subsequently, clinical impact. As we move into a future where machine learning and AI models become ubiquitous in neuroimaging research and psychopathology (Rezaei et al., 2023), it remains ever more imperative to collect high-quality data. High-volume specific training data is required in machine learning to generate accurate predictive models for biomarker discovery, precision psychiatry, and treatment-response prediction (Blair et al., 2022). More specifically, it is important to maintain the integrity of training data across diverse populations, particularly in disorders that manifest in spectra: Major Depressive Disorder, Obsessive Compulsive Disorder, Borderline Personality Disorder, Bipolar Disorder, Cannabis Use Disorder, Autism Spectrum Disorder, and healthy controls. These populations present unique challenges, including symptom severity, anxiety, attention deficits, claustrophobia, reduced tolerance for scan duration and noise, involuntary motor movement, cognitive impairments, difficulty maintaining arousal, and variability in participant engagement, which influence data quality before preprocessing begins. This paper aims to address the challenges the Brain Stimulation Lab has faced and offer recommendations for ways to operationalize and optimize the systematic collection of high-quality MRI data. While this is not an all-encompassing “gold standard,” the purpose of this article is to serve as a starting point for researchers aiming to collect large volumes of high-quality neuroimaging data from clinically diverse research participants.

The BSL sits in the scientific gap between neuroscience and interventional psychiatry. Personalized, focal transcranial magnetic stimulation (TMS) based on patient-specific MRI data is a primary research focus and treatment option available to the BSL’s clinical research participants. Given the growing number of psychiatric and neurological interventions that are dependent on neuroimaging, such as functional magnetic resonance imaging (fMRI), it is increasingly important to employ rigorous, referenced, and repeatable neuroimaging protocols to enable high-quality outcomes. In the last 10 years, the BSL has collected thousands of brain scans across over a dozen clinical trials developing novel therapeutics for psychiatric conditions. In this time, the BSL noticed minimal guidance on how to collect consistent, high-fidelity, personalizable neuroimaging data from clinical science literature.

## 2 Materials and equipment

### 2.1 Brief introduction to MRI

Magnetic resonance imaging (MRI) is a non-invasive imaging modality that combines principles of physics, mathematics, and human biology to produce high-resolution images of internal anatomy. A strong magnetic field aligns hydrogen nuclei within the body, and radiofrequency pulses temporarily perturb this alignment. As the nuclei return to equilibrium, they emit electromagnetic signals that are detected and computationally reconstructed into detailed anatomical images. For our research purposes, BSL specifically utilizes anatomical brain MRI and functional MRI for the generation of precise patient-specific neurostimulation targets.

Structural MRI provides high-resolution anatomical images of the brain and forms the foundation of most neuroimaging studies. The most commonly acquired structural sequences include T1-weighted, T2-weighted, and fluid-attenuated inversion recovery (FLAIR) imaging, each providing complementary information about brain anatomy and pathology. T1-weighted imaging provides gray-white matter contrast and is the standard sequence for assessing brain anatomy, cortical thickness, volumetric analyses, and image registration. T1-weighted images are also widely used for neuronavigation, surgical planning, and as anatomical references for functional MRI analyses. T2-weighted imaging is highly sensitive to water content and is very useful for identifying edema, inflammation, cystic lesions, and other abnormalities associated with increased fluid volume. This sequence complements T1-weighted imaging by improving visualization of pathological processes that may not be readily apparent on T1-weighted images. FLAIR is a modified T2-weighted sequence that suppresses cerebrospinal fluid (CSF) signal, improving visualization of lesions adjacent to the ventricles and cortical surface. FLAIR is commonly used clinically to detect white matter hyperintensities, demyelination, ischemia, gliosis, and other neurological disorders. At the Brain Stimulation Lab (BSL), anatomical MRI is routinely acquired to evaluate structural integrity, facilitate image registration and neuronavigation, localize functional findings, and provide a high-resolution anatomical reference for neurostimulation treatment and downstream analyses.

Functional MRI (fMRI) indirectly measures neural activity by detecting changes in blood oxygenation over time through the blood oxygen level-dependent (BOLD) signal. Oxyhemoglobin is diamagnetic, whereas deoxyhemoglobin is paramagnetic and produces faster signal decay. During neural activation, oxygen is initially consumed, resulting in a transient increase in deoxyhemoglobin, followed by a compensatory increase in cerebral blood flow that elevates oxyhemoglobin concentration and produces a measurable increase in the BOLD signal (Chow et al., 2017). Tracking these hemodynamic changes enables researchers to localize brain activity with high spatial resolution (Ekstrom, 2010). Functional MRI studies generally fall into two categories. Resting-state fMRI (rs-fMRI) measures spontaneous fluctuations in the BOLD signal while participants remain awake but do not perform an explicit task. These spontaneous fluctuations reveal intrinsic functional connectivity between distributed brain networks and are widely used to investigate network organization in both healthy individuals and clinical populations. In contrast, task-based fMRI measures BOLD signal changes elicited by specific cognitive, motor, sensory, emotional, or behavioral paradigms. By comparing periods of task performance with baseline conditions, researchers can identify brain regions associated with particular functions and evaluate how neural activation differs across individuals or disease states.

Beyond conventional structural and functional imaging, many studies incorporate specialized non-contrast MRI sequences to answer specific clinical or scientific questions. Diffusion Tensor Imaging (DTI) characterizes the diffusion of water molecules within tissue to assess white matter microstructure and structural connectivity. It is commonly used to investigate traumatic brain injury, stroke, neurodegenerative diseases, psychiatric disorders, and white matter development. Arterial Spin Labeling (ASL) is a non-contrast perfusion imaging technique that magnetically labels arterial blood water to quantify cerebral blood flow. ASL can be used to study cerebrovascular disease, dementia, epilepsy, psychiatric disorders, and treatment-related changes in brain perfusion. Time-of-Flight (TOF) Magnetic Resonance Angiography visualizes intracranial arteries without intravenous contrast by exploiting flow-related enhancement. TOF imaging is frequently used to detect aneurysms, arterial stenosis, vascular malformations, and other cerebrovascular abnormalities. Additional specialized sequences include susceptibility-weighted imaging (SWI) for detecting cerebral microbleeds, iron deposition, and venous anatomy; perfusion-weighted imaging (PWI) for evaluating tissue perfusion in stroke and tumor imaging; and magnetic resonance spectroscopy (MRS) for measuring tissue metabolites and investigating biochemical changes associated with neurological disease (Holdsworth and Bammer, 2008).

While recommendations presented in this manuscript are broadly applicable to most MRI research, including X-nuclei MRI (e.g., sodium, potassium, chlorine, and oxygen MRI); the primary focus of this paper is on anatomical and functional Proton MRI acquisition (Hu et al., 2020). Researchers employing specialized imaging sequences should work closely with imaging physicists and modality-specific experts to optimize acquisition parameters and tailor protocols to their scientific objectives. Considering that MRI data can be highly variable, the key to acquiring reliable and consistent MRI data for research purposes is to keep parameters such as the MRI scanner, field strength, task-displayed, and analysis settings consistent, which prevents differences in the interpretation of data (Jovicich et al., 2009). Furthermore, especially in fMRI data analysis, parameters involved in preprocessing data like motion correction, slice timing correction, field map correction, spatial smoothing and normalization, high-pass filters, head motion regressors, hemodynamic response, and multiple comparison correction, all influence the reproducibility of MRI data (Poldrack et al., 2017).

### 2.2 MRI settings and hardware

Given the complexity of MRI equipment, it is critical to have a thorough understanding of the available tools and to work closely with experienced MRI personnel to promptly address any issues that may arise. Each imaging facility will have its own user rules, pricing scheme, and standardized safety protocol; it is recommended to consult them while designing a new imaging study. It is imperative that researchers maintain good working relationships with the scanning facility and other research groups to support a positive academic environment. MRI system upgrades (hardware and software) are typically managed by the imaging facility, and therefore, keeping up to date to the schedule (newsletter, email, website, etc.) of these upgrades can help when executing a study. For example, at Stanford University, the BSL shares an MRI scanner with other Stanford researchers at a designated imaging facility staffed by experts in MRI physics, hardware, and imaging acquisition. The Stanford facility’s general MRI hardware along with other equipment sits in an adjacent control room. The following details the Brain Stimulation Lab’s MRI setup and details some basic equipment for MRI scanning.

### 2.3 The BSL’s MRI setup

In addition to fundamental MRI hardware, the imaging facility at Stanford University contains a range of physiological monitoring equipment such as a respiration bellows, pulse oximetry (can be used to account for artifacts in the fMRI data), skin conductance, electromyography, eye tracking (depending on the specific study design), and a video camera to monitor the participant’s face to ensure they are awake, especially during the resting-state fMRI scan. Communication with participants is enabled through a built-in intercom. For task-based functional MRI, a modular response box system integrates response device input with the MR scanner, synchronized with millisecond precision. During task-based fMRI, stimulus presentation is done via an LCD display located in the scanner room, connected to the experimental computers in the control room, and visible to the participant in the scanner through mirrors attached to the proton head coil. Additionally, to help participants see and interact with tasks in the scanner, researchers can provide MR safe corrective lenses.

Due to the strong magnetic fields produced by MR machines, it is imperative to maintain MR safety practices (Shah and Aran, 2023). To ensure patient safety while scanning, the facility has a metal detection system, ample padding and cushioning to prevent RF burns, hearing protection, and MR safe scrubs, linens, and facemasks. Participants are given an emergency alert squeeze ball before the scan, which they can use to alert research staff if they need to stop the scan for any reason, such as feeling claustrophobic, anxious, or uncomfortable. Staff regularly check in with participants via intercom, fostering a safe and comfortable clinical research environment while instructing participants during the scan.

#### 2.3.1 Fundamental MRI hardware

- MR system with various head coils
- Psychological monitoring devices (pulse oximeter, breathing belt, heart rate monitor)
- Monitors and a central MR console with appropriate software
- Display system and response devices for task-based fMRI
- Intercom and camera system to monitor the subject
- Data storage and backup systems

#### 2.3.2 MRI equipment for patient safety and comfort

- Arm, head, and body padding to prevent RF burns
- Ear protection system
- Cushioning for patient comfort
- Metal detector system
- Emergency alert system (e.g., squeeze ball)
- MR safe linens and scrubs
- MR compatible glasses for task-based fMRI
- MR safe surgical facemasks
- MR safe cleaning and organizational supplies (e.g., mops, wipes, and storage carts)

## 3 Methods

### 3.1 Recommendations for subject recruitment and participant screening

The Stanford Brain Stimulation Lab (BSL) conducts clinical trials for numerous psychiatric disorders, including treatment-resistant depression, bipolar disorder, borderline personality disorder, addiction, and obsessive-compulsive disorder. Human clinical trials with neuroimaging typically require large sample sizes to achieve adequate power, requiring a strategic, multifaceted, and organized recruitment plan. The BSL recruits participants from the community largely via media advertisements and referrals from clinics. As discussed in Figure 1, to refine the screening process, participants initially complete online surveys via REDCAP, a data management software, to ensure basic study inclusion criteria are met. Before a formal screening interview, the BSL collects medical records that are deposited to Stanford Medicine Box, a HIPAA-compliant and secure server, and requests additional information via telephone and email to confirm eligibility for study participation, such as current and past medications. During the screening process, and in accordance with flagship guidelines, e.g., from the American College of Radiology questions about MRI safety and tolerance are also asked and confirmed (Pedrosa et al., 2025). For any instances of metal in the body, the BSL uses medical records and if needed, collects data on the implant manufacturer and product serial number to verify safety^1^ ^,2^ in addition to consulting with imaging facility staff (including MR technologists and radiologists) to determine MR safety compliance (ACR Committee on Mr Safety et al., 2020). In instances where we are unable to verify safety with 100% certainty, we simply opt to exclude the subject from the study and do not perform the MR. Participants can be excluded from the study for numerous reasons, including but not limited to: unwillingness to participate in MRI, history of brain-related illness or injury that would impede MRI quality and safety, presence of any MRI-safe metals above the neck that would affect MRI data quality, presence of any non-MRI safe metals located anywhere in the body (medical and surgical implants, irremovable piercings, pacemakers, shrapnel), and history or current diagnosis of claustrophobia or significant anxiety related to MRI (Mittendorff et al., 2022). By including research participants who meet study criteria and are more likely to successfully complete scanning procedures, this recruitment process ensures participant safety while improving data quality by promoting participant adherence to study protocols, ultimately enhancing participant experience and the overall study’s success.

**FIGURE 1:**
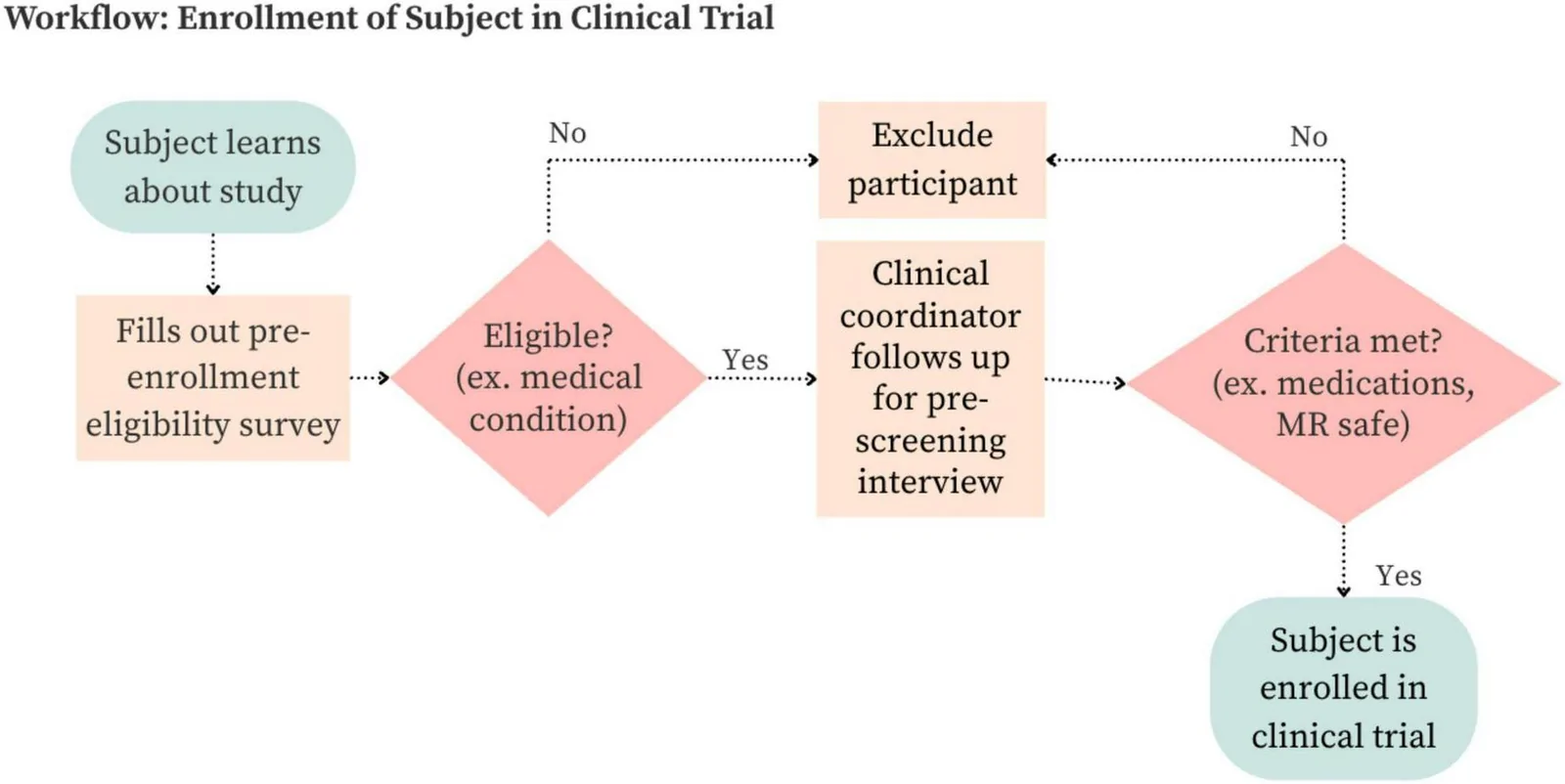
Following the patient from the first interaction to enrollment in the clinical trial.

### 3.2 MRI scanning considerations

#### 3.2.1 MRI study logistics

To avoid missing data and ensure participant safety and comfort, standardized protocols are implemented prior to and during MRI scans. The following lists strategies that the BSL employs to ensure that MRI scanning procedures run as smoothly as possible.

(1) The BSL reminds participants of their appointment the week prior and again 48 h before their MRI visit via call, text, and email. Additionally, researchers send a document detailing logistical information such as location (including precise driving and parking instructions), appointment time, and what to expect during the experimental visit to encourage punctuality.

(2) Appointments are specifically tailored to participants who travel from out of state to join the clinical trial. They are scheduled to minimize time spent traveling to be as convenient as possible, and the BSL helps arrange flights and travel accommodations. While this may not be feasible in many cases, it should be considered when recruiting for unique clinical cohorts and using similar flexible scheduling strategies can help with participant retention.

(3) Before their visit, all participants are instructed to abstain from consuming alcohol for 12 h, avoid using any recreational drugs for 48 h, wear comfortable clothing that can easily be changed out of before their scan, and remove items that may interfere with MRI scanning, including eyeglasses, jewelry, hairpins, sunscreen, and makeup. This list provides examples of items and is not exhaustive; participants are reminded to follow all MRI safety instructions provided by study staff during their visit, including Supplementary Material 2. In addition to ensuring that participants adhere to strict study protocols, these reminders help staff start scanning as quickly as possible after participant arrival.

(4) BSL staff arrive early and ask participants to meet them at the imaging center 30 min before their MRI scan to encourage readiness and to provide ample time to prepare participants for scanning procedures. During this time, participants complete MRI safety forms and questionnaires, change into MRI-safe scrubs, and provide a urine and/or saliva sample to confirm 24-h drug and alcohol abstinence. Additionally, if conducting task-based fMRI, participants familiarize themselves with tasks before scanning.

(5) After participants are prepped for MRI scanning, they are instructed on the scanning sequence, tested for any metal using a metal detector, and briefed on the scanner’s intercom and emergency alert system.

(6) For safety concerns of magnetic coil-induced heating of a closed conducting loop, participants are instructed to keep their arms and legs uncrossed during the entire scan, and arm pads are placed between their body and the scanner bore. Additionally, foam pads are placed at points of contact between body parts including arms, torso, hands, ankles, and knees.

(7) Each clinical trial has its accompanying imaging protocol, which regularly ranges from 30 to 120 min. The BSL also schedules extra MRI time to ensure that all scans are collected. This buffer gives BSL staff flexibility if participants are late or they encounter technical difficulties. In these cases, by creating breathing room in the schedule, staff can repeat scans and troubleshoot issues, thus avoiding missing data. Additionally, this extra time allows BSL researchers to complete all scans without impeding other research groups by running over their scheduled MRI time.

#### 3.2.2 MRI training and preparation

Neuroimaging techniques such as fMRI are highly sensitive to head motion (Maknojia et al., 2019). Since the BSL utilizes MRI-guided neuronavigation to offer highly personalized targeting for TMS treatment, the patient’s adherence to the MRI study protocol is essential for the precise acquisition of neural signals of interest. In addition, since MRI involves substantial financial and time investments, avoiding low-quality data is important from a cost-effectiveness standpoint. The BSL has found that the best way to mitigate potential MRI data quality issues during acquisition is to implement rigorous training and sign-off processes for all lab members working with data collection, as shown in Figure 2, along with an in-house guideline that lays out the scanning facility’s standard practices, common troubleshooting solutions, frequently asked questions, and contact information for study leads (such as what we present in this article). Figure 2 details the process by which imaging staff are trained to acquire MRI data independently. The first step involves attending a mandatory MRI safety training presentation and completing facility specific required coursework to be given access to the imaging facility. Without first attending the MRI safety presentation, staff are not allowed to continue training procedures and cannot be within the imaging facility without direct supervision. Next, new staff shadow senior researchers within the lab over the following months as they familiarize themselves with proper imaging practices. We recommend that researchers design their specific training protocols to correspond with their MRI protocol’s complexity. Clinical research staff are not MR technologists, but we recognize that The American Registry of Magnetic Resonance Imaging Technologists (ARMRIT) requires new MR technologists to complete 1000 h of supervised scanning (American Registry of Magnetic Resonance Imaging Technologists [ARMRIT], 2026) while The American Registry of Radiologic Technologists (ARRT) recommends 125 scanning sessions for candidates with radiological experience (American Registry of Radiologic Technologists [ARRT], 2024). We recognize that this step in the training protocol is more subjective to specific research groups and recommend that researchers design their training to align with expectations for staff. Once cleared by senior researchers, staff in training are tested by the facility manager and finally cleared for independent data acquisition.

**FIGURE 2:**
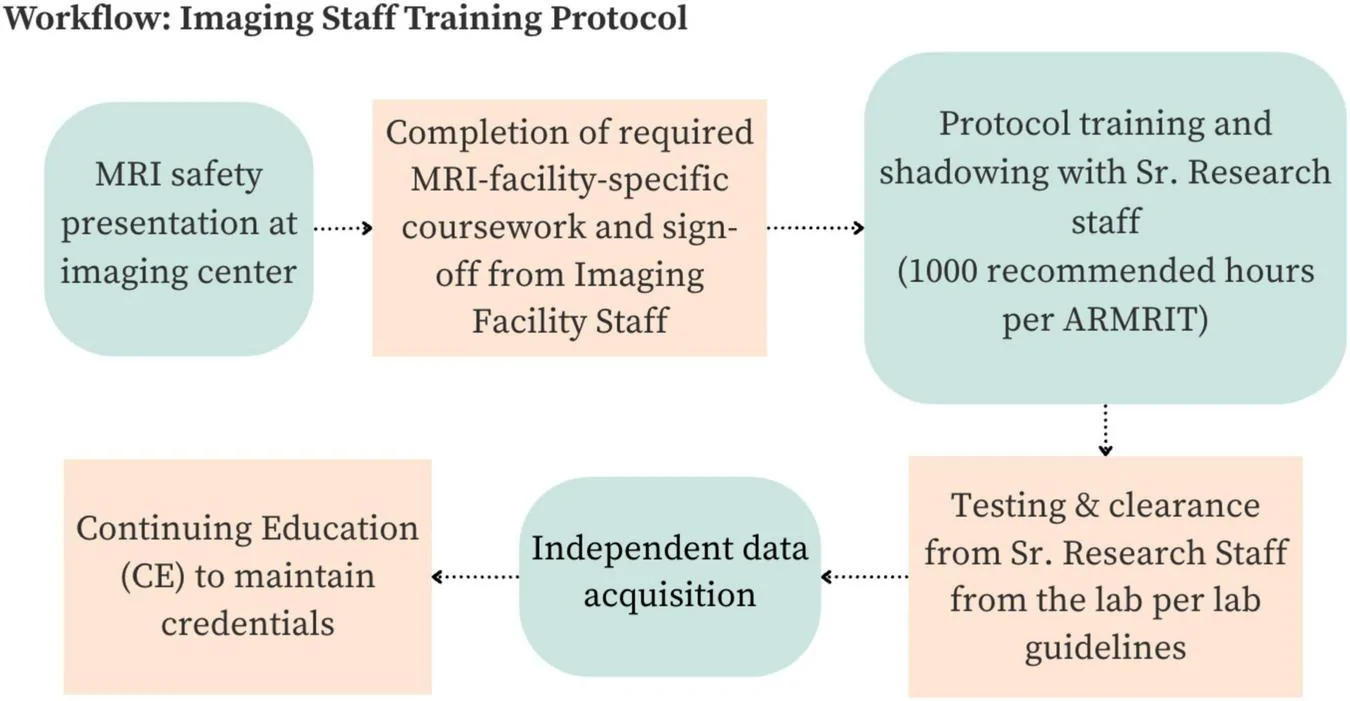
Staff training workflow for MRI.

Our standard operating procedures (SOP) addresses topics like motion in the scanner, tardiness, anxiety, and contraindications by consulting study leads or rescheduling to avoid safety risks. Excerpts of the curriculum are shown in the Supplementary Material 1. These implementations were developed by clinical research coordinators and lab members over hundreds of hours of data acquisition with diverse participants. Such a record serves as a living document and should be updated accordingly.

During MRI training, we reinforce the importance of participant safety and comfort during scanning by establishing protocols for participant setup and scanning. For participant setup in the scanner, researchers are trained to carefully apply memory foam and inflatable padding to restrict head motion and protect hearing. Researchers make sure that the participant’s head is centered and aligned to the head coil and that they are in a comfortable position before starting the scan to avoid repositioning during imaging. Research staff also provide instructions to participants to use the emergency squeeze ball if they find it necessary to stop the scan. During the scan, the participant’s alertness and head motion are continuously monitored through an in-scanner webcam. Between scans, staff maintain contact with the participant through an intercom, reminding them to keep their head still and remain awake. In our lab, checking in with participants prior to the scan and periodically during the scan has been shown to be useful in minimizing anxiety and fostering a feeling of comfort, especially for sensitive populations present in psychiatric clinical trials.

#### 3.2.3 Structural MRI, non-contrast MRI, and resting state fMRI

After setting participants up in the scanner, researchers perform preliminary scans, namely T1-weighted, T2-weighted, and FLAIR imaging, before moving to non-contrast scans and functional imaging. Participants are encouraged to keep their heads as still as possible, and research staff check in regularly between scans to ensure comfort and participant safety. They can also have their eyes open or closed during structural and non-contrast scanning, as long as they remain awake to minimize movement. DTI and structural scans have identical instructions and considerations. Once preliminary scans are completed, researchers begin resting state fMRI scanning to measure brain activity at rest. Before each resting state fMRI, patients are instructed to keep their head as still as possible, keep their eyes open, let their minds wander naturally, and try not to think about one thing in particular (Van Dijk et al., 2010).

#### 3.2.4 Task based fMRI

When incorporating tasks for neuroimaging research, particularly in fMRI studies, it is crucial to follow a detailed protocol to ensure accurate data collection and participant comfort. If the task involves personalized stimuli, such as requiring participants to take photos for visual-based fMRI tasks, it is essential to contact them to organize task-related stimuli well in advance. Additionally, the BSL allocates two to three workdays to build individual tasks and test their functionality. To ensure the participant understands the task’s instructions prior to the scan, ample time should be allotted for task practice outside the scanner. Behavioral practice should be performed on a dedicated laptop, and a mock scanner should be used for a natural setting experience. If the task requires specific equipment, such as hand grippers, they should be introduced during setup to ensure smooth task execution. Key principles for collecting task-based fMRI include allocating sufficient time for task execution in the scanner and communicating each step through the intercom using a standardized script for instructions. Ensuring that the task computer and MRI scanner are synchronized to time-lock task data accurately with the neuroimaging data is especially important for task-fMRI data collection. Additionally, it is recommended to implement a system to ensure that data is saved even if the task is ended prematurely. Finally, task-based scans should be conducted after resting-state scans to reduce head motion and avoid task-related cognitive or motor effects from carrying over into resting-state recordings. Task-based scans may involve elaborate setup and the use of response devices, which can increase head motion, while task performance itself can induce changes in functional connectivity, including within the hippocampus and dorsal attention network (Lewis et al., 2009; Tambini et al., 2010; Tung et al., 2013; Stevens et al., 2010; Lor et al., 2023).

### 3.3 MRI data analysis considerations

#### 3.3.1 The BSL’s processes to efficiently optimize MRI data quality

The quality of collected neuroimaging data is paramount. It directly determines the validity and reliability of study results, emphasizing the importance of actively ensuring high data quality. In our lab, this is especially important since the BSL uses fMRI to generate personalized functional targets for TMS treatment immediately following scanning. This requires a fast turnaround with an average of only 3 days between a participant’s baseline MRI scan and completion of data processing, including quality control, analysis, target generation, and treatment initiation. When integrated into the logistical planning, this includes re-scanning a participant as close to the scheduled time point as possible if needed.

As a starting point, the technical infrastructure of the MRI scanner is set up so that scanning acquisitions are automatically uploaded to an online platform where preprocessing and subsequent analysis gears are run (Azeez and Biswal, 2017). Our lab prioritizes data quality through a real-time quality assurance pipeline that includes visual inspections and automatically generated metrics (e.g., signal-to-noise ratio, framewise displacement) derived from MRIQC (Esteban et al., 2019) and fMRIPrep. Data with potential quality issues (e.g., ghosting, susceptibility artifacts) are flagged, and suboptimal (e.g., unacceptable skull-stripping) or failed preprocessing outputs are reprocessed for normalization, motion correction, spatial smoothing and temporal filtering during the preprocessing step shown in Figure 3 (Soares et al., 2016). In addition to reviewing the data in real-time, the BSL holds data team meetings twice a week focused on quality control (QC) of recently acquired MRI data at each stage of processing. Additionally, QC metrics from MRIQC of raw data are used to continually update a project-specific quality control dashboard, which aids in identifying potential study outliers before performing group-level analysis. Although quality thresholds are not specified in the MRIQC documentation, everyone can leverage publicly available multi-site QC outputs from OSF (Taylor et al., 2023) as a benchmark for informing data quality. To further support these QC efforts, the BSL has allocated time to develop comprehensive training materials and standard operating procedures to facilitate the onboarding of new staff members. The BSL has also established a GitHub repository where code can be easily accessed and reviewed by the team, promoting transparency and collaboration. The BSL believes that establishing and rigorously testing these workflows has been critical to our lab’s success and subsequent publications (Benrimoh et al., 2024; Batail et al., 2023; Gajawelli et al., 2024). Postprocessing, including feature extraction and hypothesis testing, occurs after quality control and analysis of pre-processing criteria. The summarized steps for image acquisition and data analysis are described in Figure 3.

**FIGURE 3:**
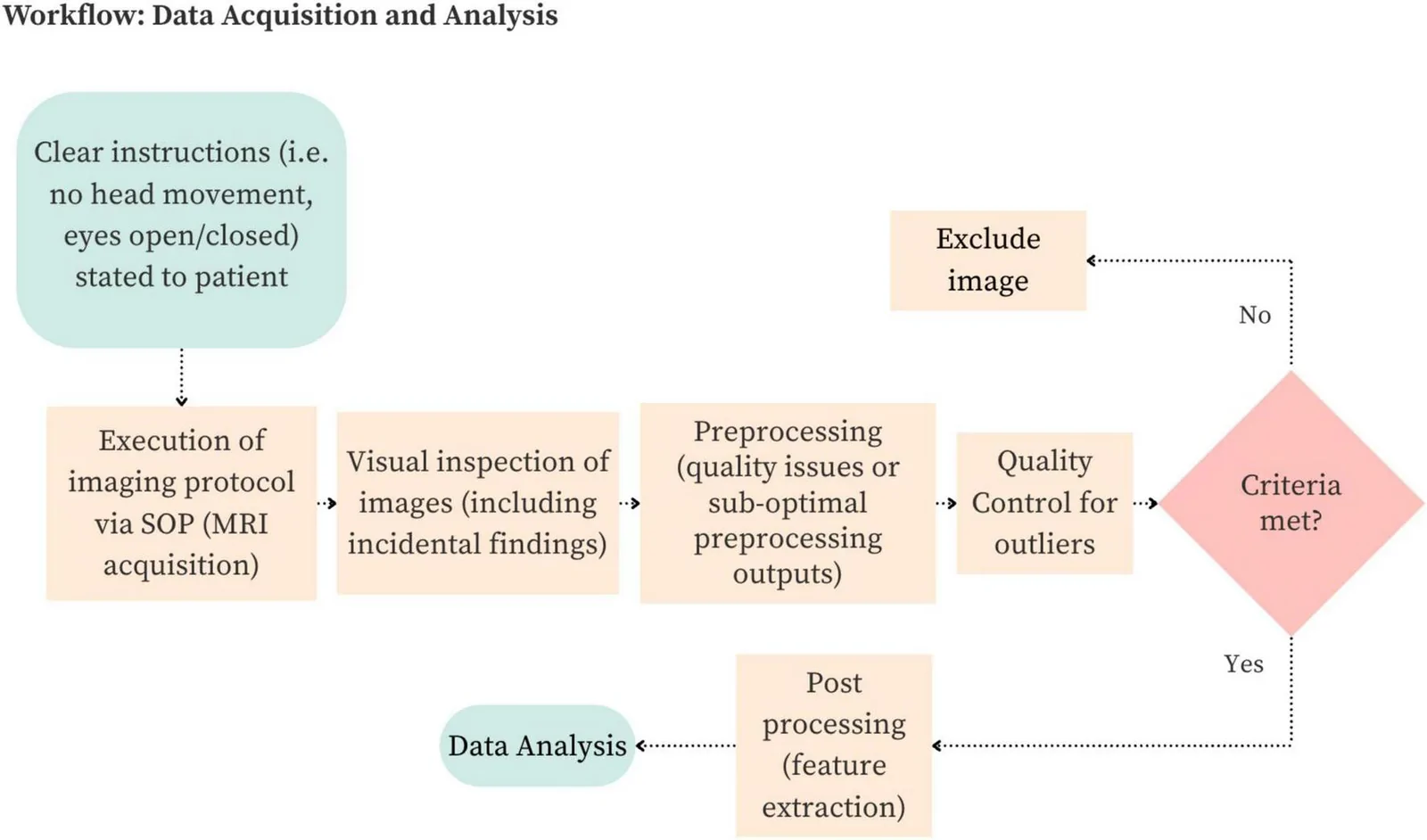
Imaging, data acquisition, and analysis process. Visual inspection of images includes ghosting, poor image quality, and incidental findings. Pre-processing includes, but is not limited to, skull-stripping, normalization, motion correction, spatial smoothing, and temporal processing. Postprocessing includes, but is not limited to, feature extraction and hypothesis testing.

In clinical study design planning, it is encouraged to consult with core facility managers and neuroimaging experts to develop a similarly rigorous QC plan and avoid imaging data loss, which can be both costly and time-consuming to manage.

#### 3.3.2 Incidental findings

Incidental findings (IF) on MRIs are common in the general population (Vernooij et al., 2007), and, therefore, present in research trials as well, making it essential to have an appropriate protocol for handling these in place, as shown in Figure 3 (Hegenscheid et al., 2013). It is important to highlight that scans acquired in a research setting are not optimized to detect potential brain abnormalities and that the research team is not professionally trained to read the acquired scans for brain abnormalities, nor responsible for finding them. In our lab, two teams are looking at the MRI scans on the day of acquisition. Firstly, scanning operators review the scans in real-time. Secondly, a data team member reviews the acquired scans as soon as they are uploaded to the online data platform. In cases where a potential brain abnormality is found, this is immediately brought up and discussed with a team of medical doctors. Subsequently, our team requests referrals from the MRI facility where the scan was acquired to a partnering radiology department. Depending on the nature of the incidental finding at a baseline scan, participation in a clinical trial may be halted until the IF is resolved. This real-time quality assessment allows the BSL to flag potential IF before the study intervention is started, thus ensuring participant safety.

### 3.4 Data storage, management, security, and governance

Large-scale neuroimaging studies generate substantial amounts of multimodal data, making robust data management practices essential for ensuring data integrity, reproducibility, and regulatory compliance. At the Brain Stimulation Lab (BSL), each participant may undergo as many as nine unique data acquisitions, including but not limited to structural MRI, resting-state fMRI, task-based fMRI, diffusion tensor imaging (DTI), resting-state electroencephalography (EEG), and TMS-EEG. Depending on the study size, these acquisitions can generate hundreds of gigabytes to several terabytes of data, underscoring the importance of standardized approaches to data organization, storage, and long-term management.

To facilitate reproducible analyses, imaging data should be organized using standardized file naming conventions and directory structures whenever possible [e.g., the Brain Imaging Data Structure (BIDS)]. Comprehensive metadata–including scanner information, operator, acquisition parameters, software versions, sequence revisions, and preprocessing pipelines–should be documented to ensure transparency and reproducibility throughout the study lifecycle. Routine documentation of hardware upgrades, software updates, and protocol modifications further facilitates longitudinal consistency and simplifies future analyses.

For multi-site neuroimaging studies, harmonization across scanner vendors, field strengths, and software versions is critical for minimizing site-related variability. Large neuroimaging consortia, including ENIGMA, ABIDE, and the Human Connectome Project, have demonstrated the importance of standardized acquisition protocols and rigorous quality assurance procedures for achieving reproducible datasets across institutions (Larivière et al., 2021; Nielsen et al., 2013; Van Essen et al., 2012). Emerging open-source frameworks, such as Pulseq, offer promising approaches for implementing vendor-neutral pulse sequences and harmonizing image reconstruction workflows, although researchers should carefully evaluate these tools based on their specific imaging requirements (Nielsen et al., 2026).

Equally important are secure data storage and governance practices. Imaging data should be securely stored immediately following each acquisition, with redundant backups maintained on institutionally approved storage systems to minimize the risk of data loss. Whenever possible, automated backup procedures and disaster recovery plans should be implemented to ensure long-term data preservation. Institutions typically provide secure research servers or enterprise storage solutions designed to support large neuroimaging datasets, and investigators are encouraged to work closely with their institutional information technology services and imaging facilities to identify storage platforms appropriate for their research needs.

All imaging and associated research data should be managed in accordance with institutional policies and applicable regulatory requirements, including the Health Insurance Portability and Accountability Act (HIPAA) (GDPR). Prior to storage and analysis, imaging data should be de-identified by removing protected health information (PHI) from DICOM headers and replacing participant identifiers with unique study identification numbers. Any linkage file connecting study identifiers to participant identities should be stored separately from research data with access restricted to authorized study personnel.

Access to research data should be controlled through institutionally managed authentication systems and role-based permissions to ensure that only approved personnel can access identifiable or study data necessary for their responsibilities. Data repositories should reside on secure, encrypted servers with routine automated backups, access logging, and audit capabilities to support institutional compliance and responsible data stewardship. Data sharing with collaborators should occur only after appropriate Institutional Review Board (IRB) approvals and, when applicable, execution of Data Use Agreements (DUAs). Shared datasets should be de-identified prior to transfer and distributed in accordance with institutional policies, sponsor requirements, and applicable regulations governing participant privacy and confidentiality.

### 3.5 Operational cost and staffing

When planning for the costs associated with collecting neuroimaging data, several key expenses should be considered, such as MRI facility hourly scan rates, imaging analysis software, data storage, data computation, and personnel costs. Staffing may include clinical research coordinators, data analysts, postdocs, and staff scientists. Data managers become increasingly important as the volume and complexity of the collected data increase. Especially in clinical trials, it is important to have a designated person or team whose goal is to ensure the quality and integrity of the data from the time of acquisition, analysis, and eventual dissemination. Data analysts may come from a wide range of backgrounds and skill sets, including but not limited to computer science, engineering, psychology, and neuroscience. When selecting a data analyst, consider their expertise in data cleaning, coding, statistics, and scientific communication. We would highly recommend considering these when writing grants and seeking justification for funding scientific endeavors.

## 4 Results

### 4.1 The BSL’s success in collecting MRI data

The BSL’s use of novel precision and personalized MRI-guided neuronavigation place them in a unique position to provide a variety of clinical populations with high treatment efficacy in a short time span (Cole et al., 2020, 2022). With this comes a high volume of valuable human clinical data requiring strict maintenance. The storage, management, quality assurance, and reproducibility of our data are important for the acute admission of treatment as well as the production of consistent, high-quality clinical research. From 2022 to 2025, the BSL has conducted more than 1,326 MRIs across numerous clinical trials, demonstrating protocol applicability in longitudinal and high-output neuroimaging studies. In these years there have only been 62 instances, representing 4.7% of scans, where MRIs were cancelled unexpectedly, as described in Figure 4. Unexpected cancellations are defined as cancellations occurring within the same week that the scan was scheduled, as last-minute cancellations incur a fee from the scanning facility and represent potential lost data. Figure 4 further elaborates a breakdown of these 62 scans, where 19 were rescheduled to later dates still within an acceptable window for data collection as per study protocol. 43 MRI scans were not completed and data was lost after scheduling, either due to last minute cancellations, scanner or facility maintenance, patient no-shows, or other extraneous circumstances. Table 1 also provides more details for scan outcomes across different patient populations. Based on our experience collecting large-scale neuroimaging data, we recommend that the following data-related needs be incorporated into research budgets and grant proposals.

**TABLE 1:** Data detailing the specific outcomes of each MRI scan from January 2022 to December 2025.

Patient population	Total MRI scans	Cancelled MRI scans	Rescheduled MRI scans	MRI data lost due to participant cancellation	MRI data lost due to MR safety concerns	MRI data lost due to imaging facility
2022–2025 MRI scan outcomes breakdown						
Major Depressive Disorder	766	28	13	12	0	3
Obsessive Compulsive Disorder	291	23	5	16	1	1
Traumatic brain injury	69	4	0	2	2	0
Healthy control	67	3	0	3	0	0
Cannabis Use Disorder	60	3	0	3	0	0
Borderline personality disorder	46	1	0	1	1	1
Bipolar affective disorder	23	0	0	0	0	0

**FIGURE 4:**
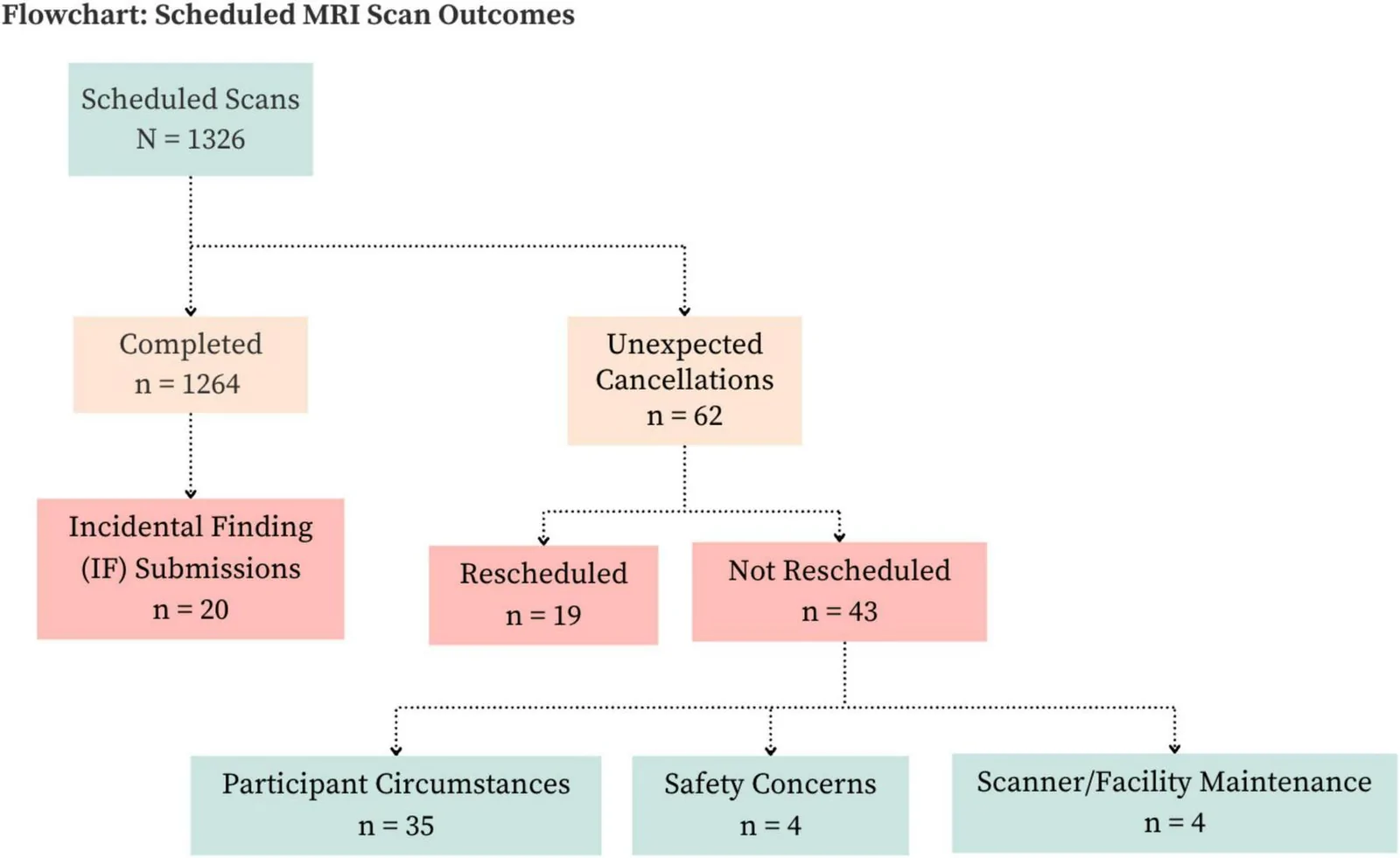
Flowchart breaking down scheduled MRI scan outcomes from January 2022 to December 2025.

Of the 62 total unexpectedly cancelled MRI scans, 19 were rescheduled for a later date while 43 scans represent lost data either due to participant circumstances, MRI safety concerns, or technical issues at the scanning facility.

### 4.2 Special considerations for clinical populations

The Brain Stimulation Lab runs multiple clinical trials involving many diverse clinical populations including patients with Treatment Resistant Major Depressive Disorder, Traumatic Brain Injury, Obsessive Compulsive Disorder, Cannabis Use Disorder, Bipolar Disorder, Borderline Personality Disorder, and healthy controls. Accommodating patients is crucial to successfully collect imaging data. It is important for researchers to identify when certain patient populations or specific research participants require extra assistance during scanning procedures.

A common challenge for all patient populations is claustrophobia and general anxiety related to scanning. Asking participants during study recruitment if they are claustrophobic, dedicating extra time for participants to prepare and acclimate to the MRI machine, and checking in regularly during scanning will help researchers collect imaging data more effectively. Additionally, researchers should make unique considerations for specific clinical populations. Major Depressive Disorder is often characterized by fatigue, difficulty concentrating, and loss of interest in activities, among other symptoms (American Psychiatric Association, 2022). As a result, research participants may have difficulty staying awake in the MRI, focusing on MRI tasks, or being motivated to come to study visits. To encourage participants with MDD, research staff at the BSL remind them of appointments, keep in regular contact with them during scanning procedures, and monitor participants closely to ensure that they stay awake. Scanning participants with Obsessive Compulsive Disorder, characterized by unwanted obsessive thoughts and compulsive behaviors (American Psychiatric Association, 2022), poses a different set of considerations for research staff. Participants with OCD may need extra time scheduled before or during scanning procedures depending on their unique obsessions or compulsions, so it is important to thoroughly screen participants to best accommodate them and make them feel comfortable during their MRI visit. The BSL would often reserve up to 1 h of extra time before scanning to ensure that participants were not rushed and could easily begin scanning procedures. Similarly, researchers should consider challenges that their patient populations may face related to MRI and proactively address them to increase adherence to study protocols and minimize lost data due to last minute cancellations, running out of time to complete scanning procedures, or needing to terminate the scan prematurely.

## 5 Conclusion and discussion

Human neuroimaging data acquisition can be logistically challenging, expensive, and often noisy, yet it offers a rich opportunity to advance our understanding of human neurobiology, explore non-pharmacological treatments, and make lasting clinical impacts on psychiatric patients. The Stanford Brain Stimulation Lab operates at the intersection of the field of translational neuropsychiatry and advances in neuroimaging technology, collecting hundreds of sessions of neuroimaging data per year across a wide range of neuropsychiatric disorders. While running multiple clinical trials involving neuroimaging data acquisition, the BSL observed a gap in scientific literature for guidance on the collection of consistent, high fidelity neuroimaging data.

This literature gap has become increasingly important as neuroimaging datasets continue to scale. Over the past 30 years, the influx of neuroimaging data has enabled the application of novel data science techniques to neuroimaging datasets, presenting the opportunity to address increasingly complex scientific questions. Adherence to best practices in data collection and management can maximize the quality and reliability of these imaging datasets, ensuring robust findings and advancing scientific knowledge.

In this article, we provide operational recommendations and scalable protocols for researchers interested in conducting similar scientific research. Recommendations include, but are not limited to, extensive training of personnel, meticulous calibration of equipment, rigorous real-time quality control measures, detailed documentation (SOP, hardware, software logs), data handling, subject recruitment and establishing consistent workflows. High-quality imaging data in human clinical research trials demand careful attention to detail and a commitment to scientific rigor. Despite its strengths, these protocols may require substantial institutional resources, including access to MR equipment, a diverse set of trained personnel, and secure data storage. Smaller groups may need to adapt components of the protocol to better meet their needs. As neuroimaging becomes increasingly central to translational neuroscience discovery and its expanded fields, including interventional psychiatry and precision medicine, we hope that the recommendations outlined in this article increase the volume of imaging datasets, ensure data integrity and reproducibility, and advance our understanding of the complexities of human neurobiology.

### 5.1 Limitations

The recommendations presented here are intended to improve the quality, consistency, and reproducibility of neuroimaging data acquisition; several important sources of variability remain beyond the control of individual research groups. MRI data quality is inherently influenced by differences in scanner hardware, field strength, gradient performance, radiofrequency coils, vendor-specific pulse sequence implementations, software upgrades, and image reconstruction algorithms. Even when standardized acquisition protocols are employed, these technical differences may introduce variability across scanners, institutions, and longitudinal studies.

Subject related factors also contribute substantially to data variability. Clinical pathology, medication, fatigue, anxiety, head motion, cognitive impairment, and fluctuations in participant engagement can all affect image quality despite careful preparation and standardized operating procedures. While many of these challenges can be mitigated through thoughtful study design and with the aid of experienced research personnel, they cannot be completely eliminated, regardless it is helpful to know the factors that may be out of your control.

Lastly, the operational workflows described in this manuscript reflect the experience of a single high-volume academic neuroimaging center and have been refined through years of iterative implementation across thousands of scans. Although many of the recommendations are broadly applicable, individual institutions may need to adapt these practices to accommodate differences in scanner infrastructure, staffing, institutional policies, patient populations, and available resources. We do not attempt to provide exhaustive guidance on sequence optimization, MRI physics, image preprocessing, or statistical analysis, as these topics are extensively covered elsewhere in the neuroimaging literature. Rather, our goal is to complement existing methodological guidance by sharing practical strategies that support the acquisition of high-quality, reproducible neuroimaging data in clinical research settings.

## Data Availability

The data analyzed in this study is subject to the following licenses/restrictions: Due to the privacy concerns surrounding psychiatric patient data, our Institutional Review Board (IRB) mandates individualized review before data sharing. We have generated anonymized data relating to our research findings and are prepared to share it with scientists who have established research and data protection protocols consistent with Stanford University guidelines. Requests to access these datasets should be directed to AA, aazeez@stanford.edu.

## Footnotes

1
www.MRIsafety.com

2
www.mriquestions.com
