# Supplementary material for: A practical framework for large-scale neuroimaging data collection in clinically diverse populations: experience from the Stanford Brain Stimulation Lab
Source: Front Neurosci. 2026 Oct 5;20:1849837. doi: 10.3389/fnins.2026.1849837 (PMC13677880; doi:10.3389/fnins.2026.1849837)
Supplement: Supplementary file 1 [file Data_Sheet_1.pdf]

Supplement 1: Excerpts from our in-house curriculum on common issues that may arise when running an MRI, and how to respond.

Common obstacles you should become familiar with resolving during MRI operator training:

<u>What to do if you run out of time due to Center for Neurobiological Imaging (CNI) equipment malfunction</u>

- Do not run over scheduled time slot
- Follow study-specific priority of scans as advised by thePrincipal Investigator, Program Director, or their designee, as appropriate.
- Inform the MRI Research Facility Manager of the technical issue that occurred, and they can book a new slot at no extra cost.

<u>Common interruptions during scan</u>

- Participant squeezes emergency squeeze ball
  - **During scan**
    - Stop scanning
    - Immediately ask the participant over intercom if they are okay
    - Calmly remove them from the scanner if they are not able to continue
      - Follow emergency procedures as needed
    - If they are all right to continue, restart the sequence when ready
    - If squeezing the ball for a non-emergency, explain to them that for non-urgent concerns, they can let us know between scans over the intercom, when scan operator checks in with them
  - **Before/between scans**
    - Ask participant over intercom if they are all right
    - Pull them out if they are not able to continue
      - Follow emergency procedures as needed
    - If they are all right to continue, restart the sequence when ready
    - If squeezing the ball for a non-emergency, explain to them that for non-urgent concerns, they can let us know between scans over the intercom.

- **Display cable errors & the screen in the scanner room does not display desired stimuli**
  - **During scan**
    - Stop scan
    - Let participants know that there has been a technical issue, and that frequent updates will be provided.
    - Check cable connection stability
      - Unplug & replug
    - Check console is configured to display from the desired source
    - Make sure the display monitor in the MRI room is turned on, functioning properly
    - Ask participant if they are able to see anything displayed in front of them
  - **Before/between scan**
    - Let participants know that we had a technical issue and that frequent updates will be provided.

        - Check cable connection stability
            - Unplug & replug
        - Check console is configured to display from the desired source
        - Make sure the display monitor in the MRI room is turned on, functioning properly
        - Ask participant if they are able to see anything displayed in front of them
- Scan encounters an error, does not start scanning
    - Inform participants that there is a technical issue, their safety is not at increased risk, and that frequent updates will be provided.
    - Try a Transceiver Processing and Storage reset (unique to GE MRI systems) or other software level reboot
        - If that doesn't work, try rebooting scanner
- Operator starts a sequence erroneously/without prepping scan/in wrong order
    - Stop scan
    - Let participants know that we'll be starting the next sequence shortly, repeat instructions as needed.
    - Redo calibrations and shim as needed

<u>Common compliance, safety, eligibility issues</u>

- **Participant does not show up/does not get hold of you when late/gets lost**
    - Confirm Outlook calendar and CNI Scheduler for correct start time
    - Confirm with study staff that they confirmed the MRI visit with the participant
    - Call / text / email participant using contact info on REDCap
        - If they don't reply/pick-up, leave a voicemail
    - If they are a no-show:
        - Cancel remaining CNI Scheduler booking if possible
        - Call / text study staff to let them know they did not show up, will need to reschedule as appropriate

- **Participant shows up too late and there isn't enough time to run the scans needed**
    - Meet them in person
    - Let participant know that, due to time limits, we will not be able to complete the MRI scan.
        - Ask them to reschedule with study staff at their earliest convenience
    - Cancel remaining CNI Scheduler booking if possible
    - Call / text study staff to reschedule as appropriate

- **Participant expresses/shows anxiety about scan/claustrophobia**
    - Before scan
        - Explain scan's general workflow and timeline
        - Let them know that claustrophobia/anxiety related to MRI is not uncommon, and that we can make adjustments to ensure participant comfort and safety
        - Reassure them that if they need to stop the scan and come out, they can always use the squeeze ball to let us know to promptly stop the scan
    - During scan

        - Ask them if they are doing okay before each sequence, be as reassuring as possible
        - Remind them of the sequence durations while giving instructions
        - If/when needed, ask the Principal Investigator or their designee which scans should be prioritized

- **Participant has potential contraindication/intolerance to MRI procedure**
    - Do not allow participant to go into MRI scanner until issue is resolved
        - Do not take any risk to participant safety --> do not proceed with a scan until the issue is resolved.
        - Immediately alert supervisor and/or Principal Investigator as needed to confirm safety before starting scan sequence
        - Check with study staff whether there may have been a potential contraindication to MRI that was not already documented in records
        - Ask participant to confirm safety with their physician/health care provider. Study physician may request participant to acquire and forward medical records to be emailed or faxed for study physician review for confirmation.

- **Participant has accompanying family/visitors who are curious about MRIs**
    - They are not allowed to enter the MRI locked area without proper MRI screening
    - They are allowed to wait in the lobby
    - If a participant is unable to continue scanning without assistance from family/visitor, they will need to let study staff know prior to the MRI visit.
        - Anyone who needs to enter the MRI room must first undergo screening (same procedure as participant) prior to entering the scanner and take the same safety precautions.

- **Participant wants a copy of their scan/has questions or concerns about something medically relevant about their brain**
    - Study staff will not provide any medical advice or instruction
    - If appropriate, study staff will inform study participants that the scan is not suitable for diagnosis, instead it is only used for research purposes
    - Copies of the MRI scan or screenshots of the brain will not be provided
    - If concerns persist, study participant will be instructed to contact their primary care physician or relevant health care providers.
    - Abnormal findings will be escalated to study physician, as appropriate

- **Participant weight causes issues / does not fit into the bore of the magnet**
    - Magnet weight limit is 350 lbs.
    - Participants are not allowed to be scanned without permission from CNI staff if >350 pounds due to concerns about scanner safety.
    - Participants too light --> can use a different localizer scan (Gradient vs. Spin echo)

- **Participant falls asleep during the scan**
    - Do not interrupt scan
    - Remind participant to try their best to stay awake for the duration of the scans after current scan is over
    - If time allows, consider repeating the scan

- **Participant moves around or talks during the scan**
    - Do not interrupt scan
    - Remind participant to try their best to stay still and quiet for the duration of the scans after current scan is over
    - If time allows, consider repeating the scan

- **Participant says they want to use the restroom during the scan**
    - Stop scan
    - Ask participant if they need to come out now, or if they can wait until end
    - If need to come out now
        - Take participant out of scanner
        - Re-align participant in scanner
        - Re-run localizers
        - Restart scan & calibration/shim as needed
    - If they can wait
        - Restart scan & calibration/shim as needed

General strategies to resolve technical problems

- Learn how to perform a Transceiver Processing and Storage reset if using GE MRI
- Learn how to reboot scanner
- Wait to prescribe / save prescription for next sequence until previous one is complete
- Check that participant can hear you before starting any scans
- Check that participant can see the fixation cross clearly before starting scans
- Confirm that all needed cables are plugged in for triggering stimulus presentation, software, and other experimental equipment
- Contact supervisor / CNI staff if additional help is needed
