# Supplementary material for: A practical framework for large-scale neuroimaging data collection in clinically diverse populations: experience from the Stanford Brain Stimulation Lab
Source: Front Neurosci. 2026 Oct 5;20:1849837. doi: 10.3389/fnins.2026.1849837 (PMC13677880; doi:10.3389/fnins.2026.1849837)
Supplement: Supplementary file 2 [file Data_Sheet_2.pdf]

Supplement 2: Stanford Lucas Center for Imaging MRI safety form.

MRI PRE-EXAMINATION SCREENING FORM

Richard M. Lucas Center for Imaging
Department of Radiology, Stanford University School of Medicine
1201 Welch Road, MC 5488, Room P280, Stanford, CA 94305-5488
(650) 521-7836, (650) 723-7577

Date ________________________ **Magnet ☐3T1 ☐3T2 ☐3T3 ☐7T**

Name ____ Height__________ Weight__________
Last name First name M.I.

Date of Birth ____________________ ☐Female ☐Male Ethnic Origin ______________________

Address____ City______________________________

State______ Zip Code__________ Phone (H)(_______)____________ (W)(_______)______________

Physician's name & address____

1. Have you ever had surgery or other invasive procedures? ☐Yes ☐No If yes, please list below. Use back if needed.
   Type:____ Date: _______/_______/_______
2. Have you had any previous MR studies? ☐ Yes ☐ No If yes, please list the most recent below.
   Area of Body Date Facility Name & Location
   ____________________ _______/_______/_______ ______________________________
3. Have you ever had an injury to the eye(s) by a metallic object (metallic slivers, shavings, or foreign body)? ☐Yes ☐No
4. Are you or think you may be pregnant, experiencing a late menstrual period, or having fertility treatments? ☐Yes ☐No
5. Are you currently taking or have recently taken any medication? ☐Yes ☐No Please list:____________________
6. Do you have drug allergies, or have you had an allergic reaction? ☐Yes ☐No Please list:____________________
7. Have you ever had an allergic reaction to a MR/CT/X-Ray contrast media injection? ☐Yes ☐No
8. Do you have or previously had kidney problems and/or diabetes? ☐Yes ☐No Please list:____________________
9. Endoscopy or colonoscopy in the last three months ☐Yes ☐No
10. Have you taken sedation today? ☐ Yes ☐ No
11. Are you wearing a Glucose Monitor? ☐ Yes ☐ No * **If yes, remove it before entering the scan room**.

Some of the following items may be HAZARDOUS to your safety & some may interfere with the MRI exam.
Do you have any of the following:

☐Yes	☐No	Cardiac pacemaker or defibrillator	☐Yes	☐No	Pessary or bladder ring
☐Yes	☐No	Internal cardiac pacing wires	☐Yes	☐No	Transdermal drug delivery patch **(Remove)**
☐Yes	☐No	Heart valve prosthesis	☐Yes	☐No	Prosthesis (eye, penile, etc.)
☐Yes	☐No	Aneurysm clip or brain clip	☐Yes	☐No	Artificial limb or joint
☐Yes	☐No	Carotid artery vascular clamp	☐Yes	☐No	Electrodes (on body, head or brain)
☐Yes	☐No	Neurostimulator or DBS	☐Yes	☐No	Shrapnel, buckshot, or bullets
☐Yes	☐No	Spinal fusion stimulator	☐Yes	☐No	Metal fragments (eye, head, ear, skin)
☐Yes	☐No	Implanted drug infusion device	☐Yes	☐No	Metal or wire staples, sutures, mesh implants
☐Yes	☐No	Insulin pump	☐Yes	☐No	Metal rods in bones; joint replacements
☐Yes	☐No	Venous umbrella	☐Yes	☐No	Implant or dentures held in place by a magnet
☐Yes	☐No	Tissue expander (breast, etc.)	☐Yes	☐No	Bone or joint pin, screw, nail, wire, plate
☐Yes	☐No	Aortic or vascular clips	☐Yes	☐No	Wig, toupee, or hair implants, extensions
☐Yes	☐No	Cochlear, otologic or ear implant	☐Yes	☐No	Circle/Big Eye or Triggerfish Contact Lenses
☐Yes	☐No	Stents, filters, or coils (vascular or other)	☐Yes	☐No	Tattoos: body, eyeliner, eyebrows or lips
☐Yes	☐No	Shunt (spine or ventricles)	☐Yes	☐No	Acupuncture beads or needles ***(Remove)***
☐Yes	☐No	Vascular access port or catheters	☐Yes	☐No	Body/head piercing(s) ***(Remove before scan)***
☐Yes	☐No	Swan-Ganz catheter	☐Yes	☐No	Hearing aid ***(Remove before scan)***
☐Yes	☐No	Harrington rods (spine)	☐Yes	☐No	Dentures ***(Remove before scan)***
☐Yes	☐No	Intrauterine Device (IUD)	☐Yes	☐No	Seizures or motion disorders

PLEASE REMOVE ALL athletic clothing and undergarments with antimicrobial solution in the material.
***PLEASE REMOVE ALL METAL OBJECTS* before MR exam including: Bra, Cell Phone, Keys, Hair Pins & Barrettes, Jewelry, Watch, Safety Pins, Paperclips, Money Clip, Coins, Pens, Pencils, Belt, Pocket Knife, Clothing with Metal or Metal Buttons. *HEARING PROTECTION* is required during the MRI exam.**

Signature of Person Completing form ______________________________**Date**____________________

Form Completed by: ☐ Patient / Volunteer__________________ ☐ Relative:______________________
☐ Physician:______________________ ☐ Other:______________________

Form Reviewed by (please PRINT NAME clearly & attach to consent form):________________________

Office use Only

2nd on-call person: Cell Number: ______________ **Name:** ______________________ **Location in Lucas Building:** __________
